# Supplementary material for: Quantitative measurement of antibiotic resistance in Mycobacterium tuberculosis reveals genetic determinants of resistance and susceptibility in a target gene approach
Source: Nat Commun. 2024 Jan 12;15:488. doi: 10.1038/s41467-023-44325-5 (PMC10786857; doi:10.1038/s41467-023-44325-5)
Supplement: Supplementary file 1 — Supplementary Information [file 41467_2023_44325_MOESM1_ESM.pdf]

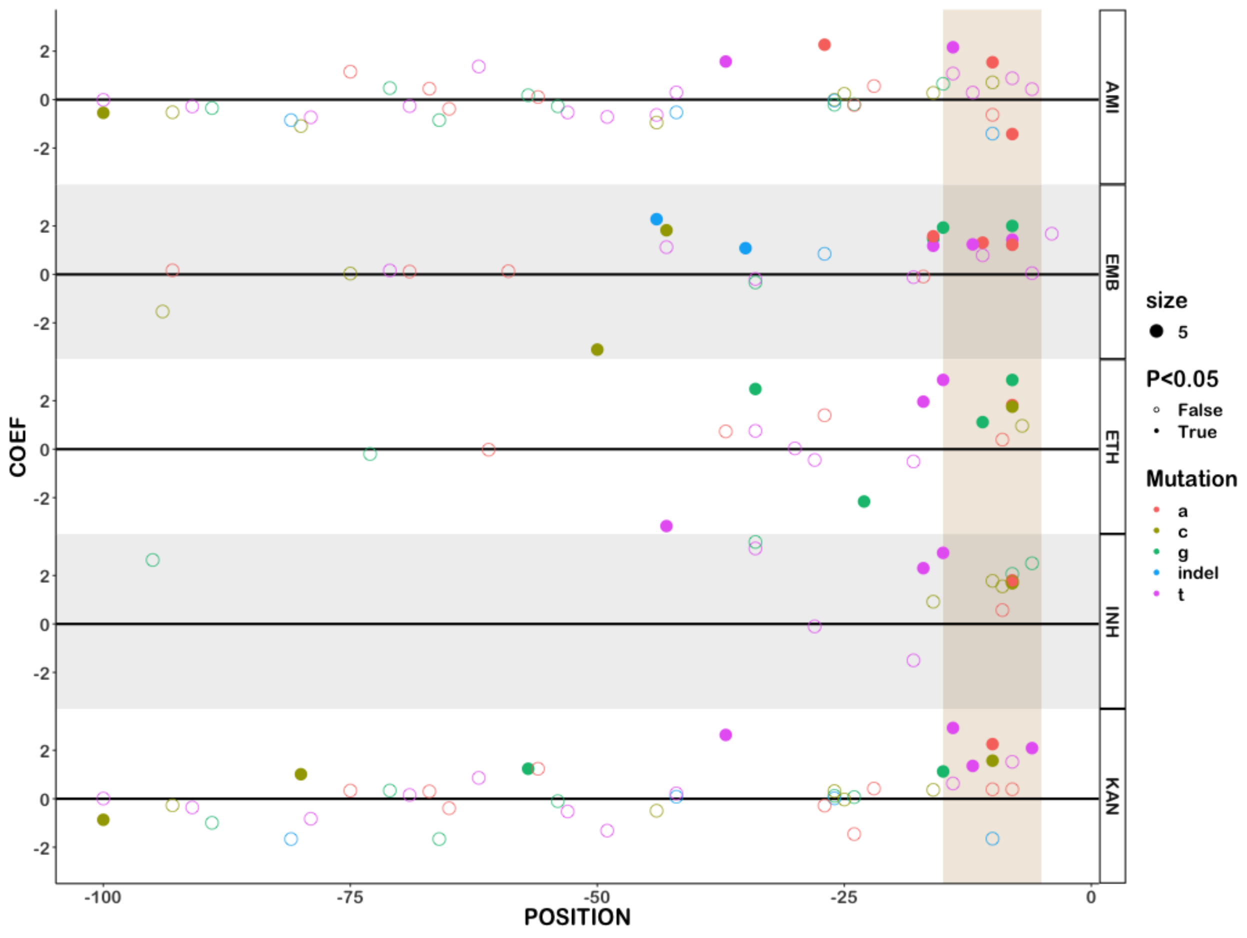

Figure S1: Resistant promoter mutations are enriched near the -10 element. Promoter mutations are shown by position and colored by the mutant nucleotide. Mean effects of mutations with significant effects ($p<0.05$) after Benjamini Hochberg correction are shown in solid circles. The -15 to -5 region is highlighted in tan. For all drugs, all mutations in the 100bp directly upstream of each gene considered by the model for that drug was included. Sample sizes and exact p-values for data shown are listed in Supplementary Data 3.

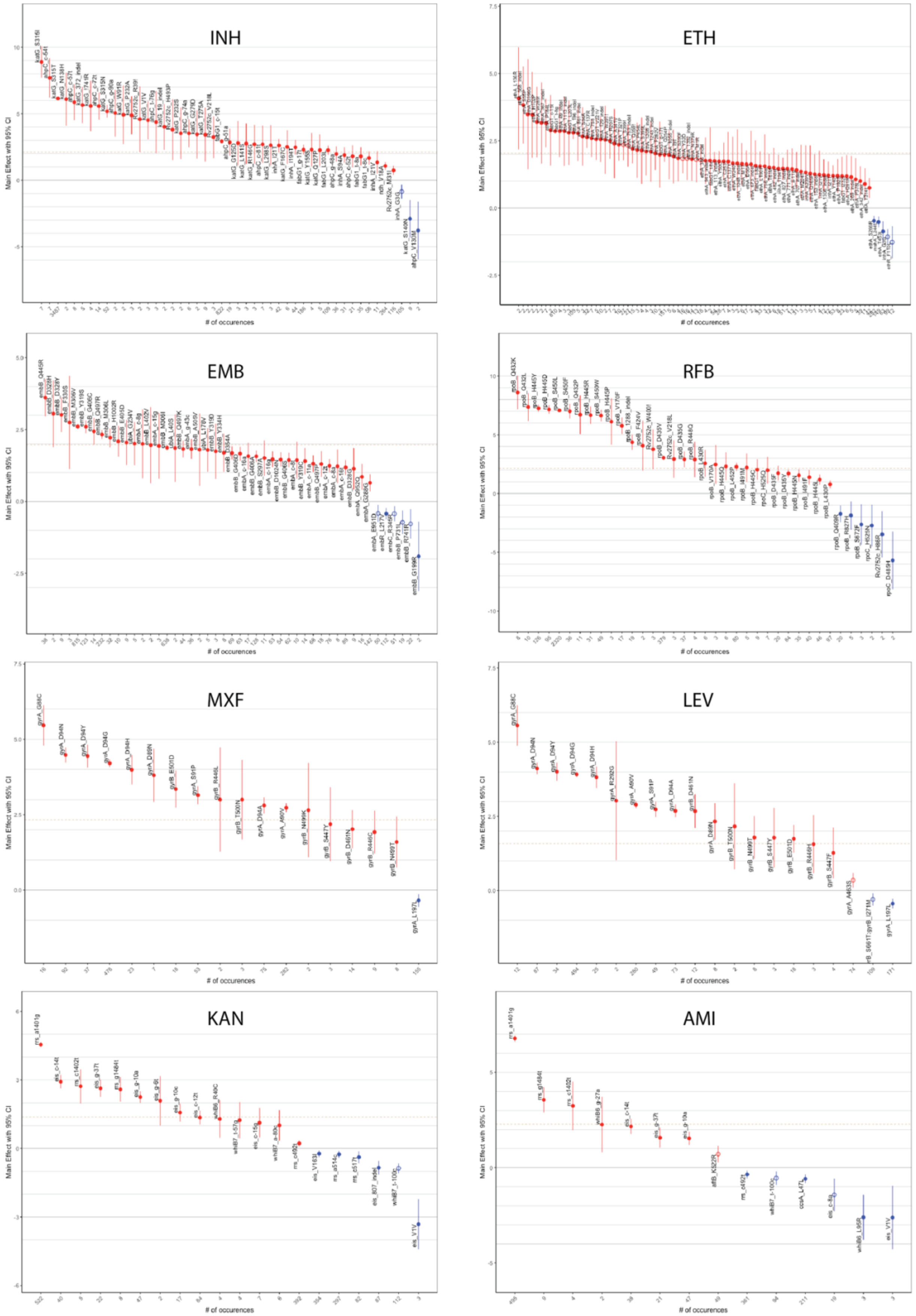
INH
ETH
EMB
RFB
MXF
LEV
KAN
AMI
Main Effect with 95% CI
# of occurences

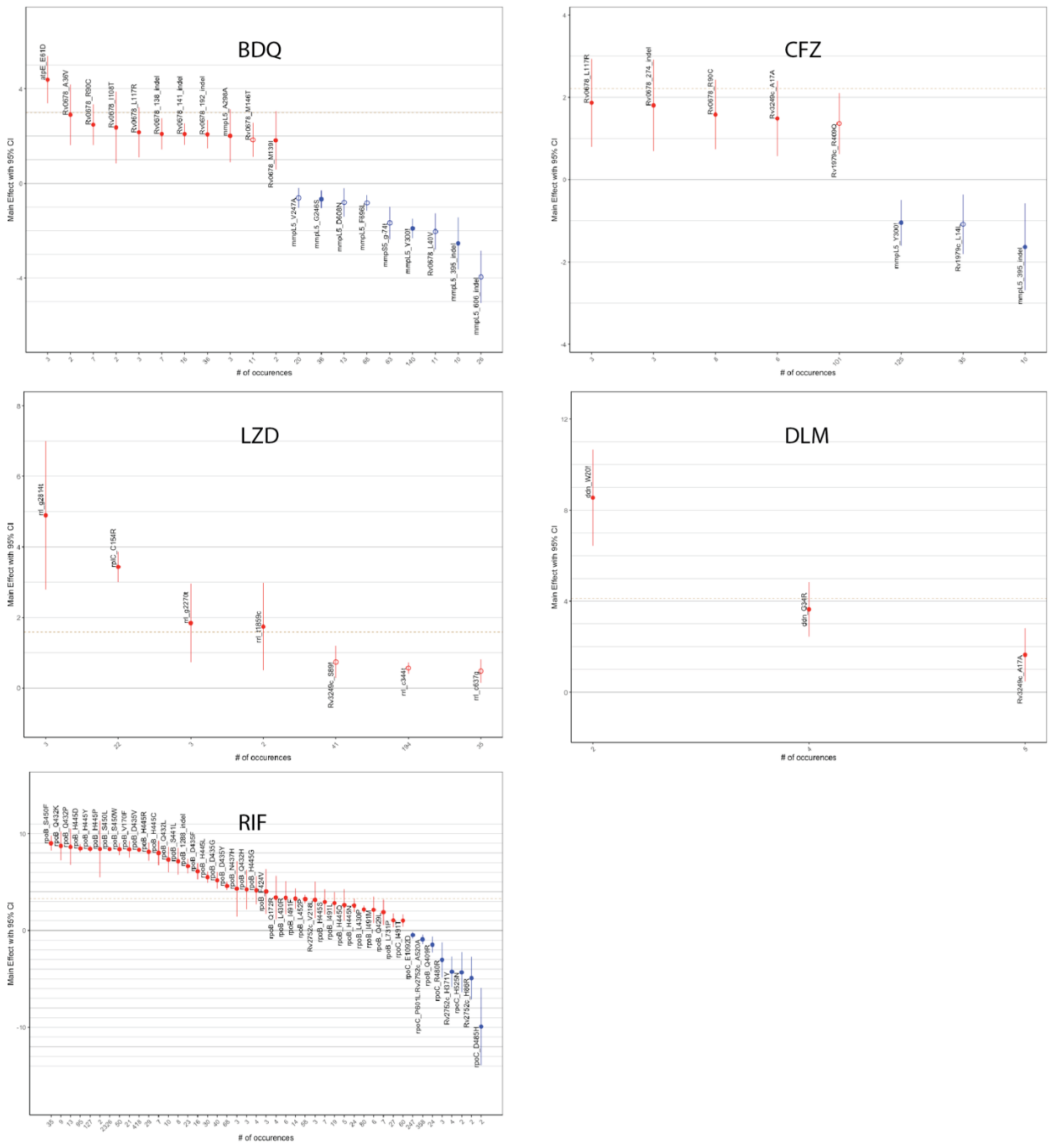

Figure S2: Effects of common (n>10) and/or homoplastic mutations on 13 anti-tuberculosis drugs. Homoplastic mutations are solid circles and ECOFFs (minus baseline MICs) are shown as tan lines. Error bars represent mean effect +/- 95%

confidence interval. Sample sizes and exact p-values for data shown are listed in Supplementary Data 3.

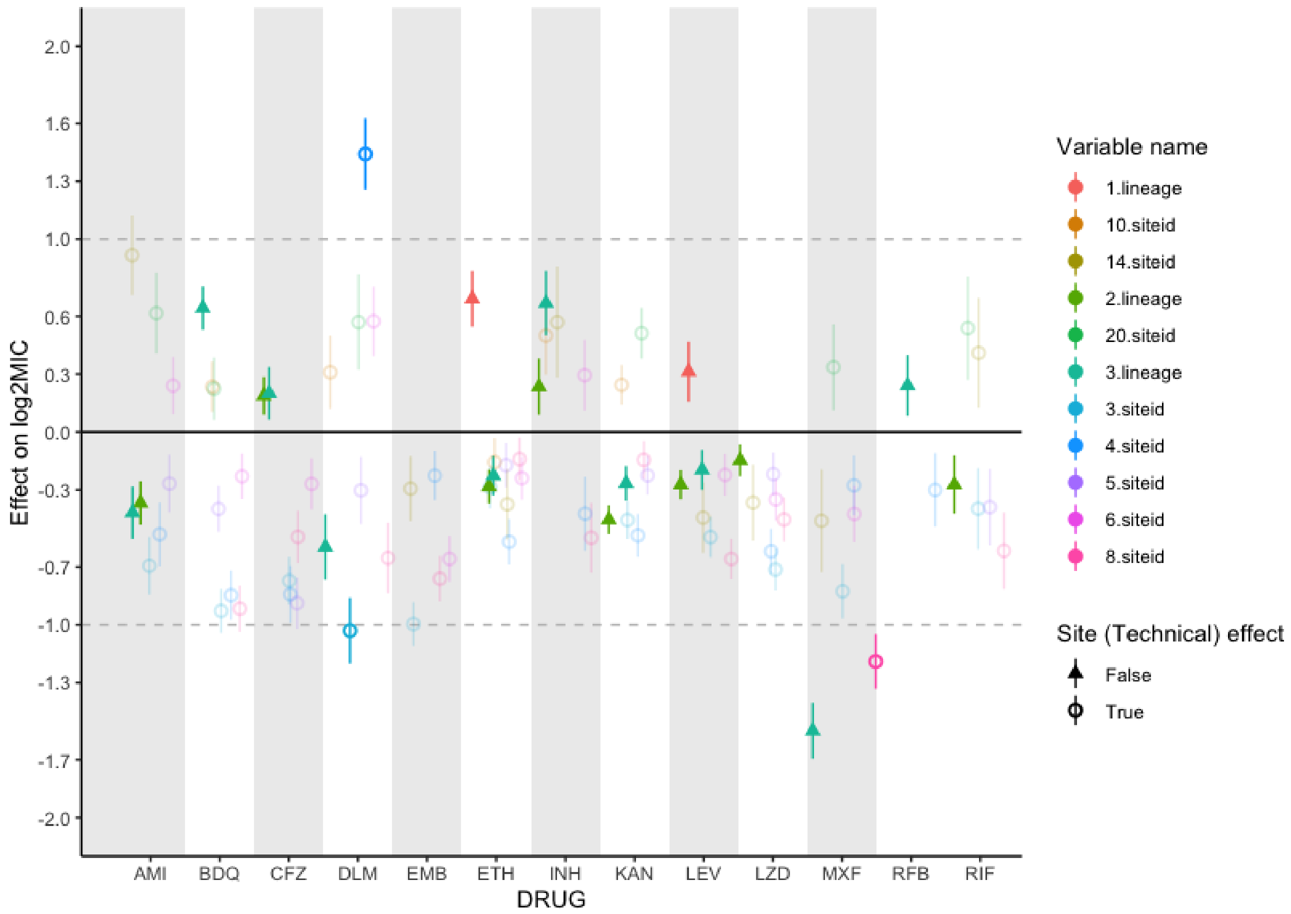

Figure S3: Effects of lineage and site (technical variability) on MIC. Significant effects of lineage and site of phenotyping on MIC compared with lineage 4 and site 2 as reference. No significant effects were identified for lineage 6. Points are mean effect on MIC with error bars highlighting the 95% confidence interval. Sample sizes and exact p-values for data shown are listed in Supplementary Data 3.

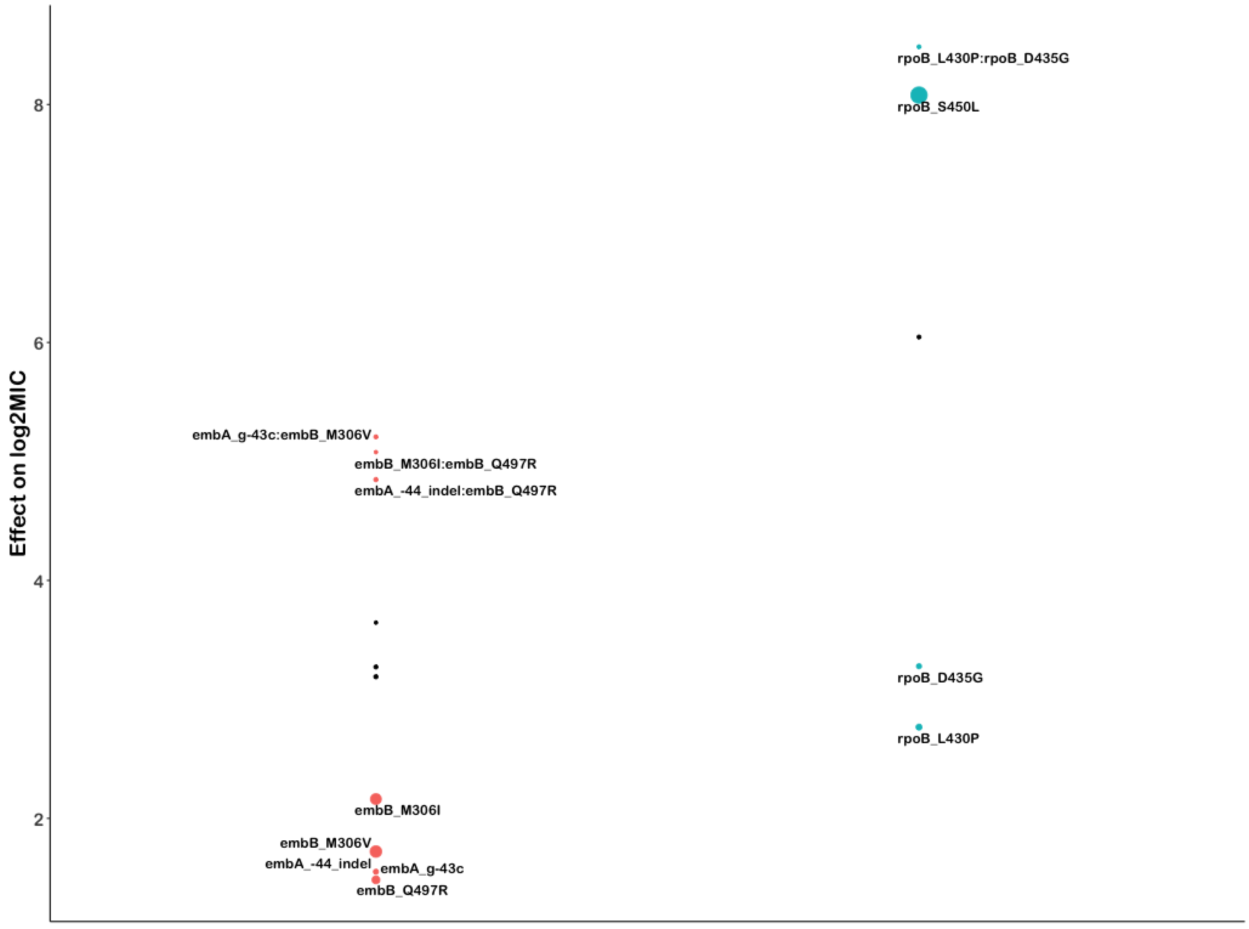

Figure S4: Mutation pairs cause interaction beyond additivity in ethambutol and rifampicin. Individual, predicted additive (black), and actual combined mean effects of mutation pairs on log2MIC. Data from this figure are available in table S10. Common single resistance mutations are provided for reference.

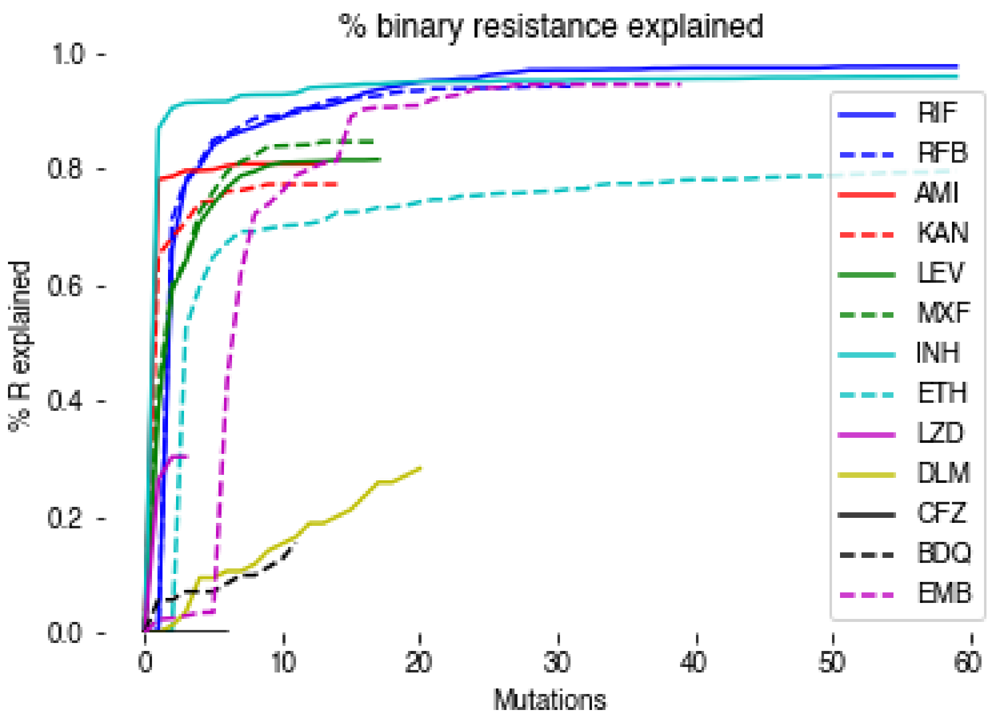

Figure S5: Heritability of binary phenotype for 13 anti-tuberculosis drugs. The proportion of binary resistance explained by each additional mutation is shown by drug. Curves asymptotically approaching horizontal indicate that few major mutations in catalog genes remain to be described.
