# Supplementary material for: Quantitative measurement of antibiotic resistance in Mycobacterium tuberculosis reveals genetic determinants of resistance and susceptibility in a target gene approach
Source: Nat Commun. 2024 Jan 12;15:488. doi: 10.1038/s41467-023-44325-5 (PMC10786857; doi:10.1038/s41467-023-44325-5)
Supplement: Supplementary file 3 — Description of Additional Supplementary Files [file 41467_2023_44325_MOESM3_ESM.pdf]

Description of Additional Supplementary Files:

Supplementary Data 1: Total high quality samples and proportion of resistant samples by drug. Intermediate and resistant cutoffs are from Fowler et al. 2021

Supplementary Data 2: Number of isolates with 1-10+ nonsynonymous mutations in target genes by drug.

Supplementary Data 3: No-interaction model results for 13 drugs in this study. All MICs are reported in log2 space. Coefficients represent mean effect on MIC in censored interval regression model. 95% confidence intervals are also provided. Mutations that have significant effects (BH_CORRECTED_P<0.05) after Benjamini-Hochberg correction are highlighted in green.

Supplementary Data 4: Promoter mutations are associated with significantly different effects on MIC for INH, EMB, and KAN. Beta and pvalues of a linear regression test are shown for the effect of a promoter mutation on log2(MIC) when controlling for gene. Fisher exact odds ratios (Promoter 0/1 vs significant MIC elevating status, two tailed)

Supplementary Data 5: Homoplasy predicts MIC-elevating behavior of mutations for most drugs. Fisher exact odds ratios and P-values (two tailed).

Supplementary Data 6: Proportion of mutations associated with subECOFF MIC elevations by drug

Supplementary Data 7: Effects of additional mutations for embA and gyrB mutants in EMB, LEV, and MXF. Counts, mean, and median MIC for each subgroup are shown. Additional mutations present in embB (EMB) or gyrA (LEV,MXF) appear in the additional mutations column.

Supplementary Data 8: Matched mutation-phenotype associations with 2021 WHO tuberculosis catalogue. All CRyPTIC MIC values are derived from Supplementary Data 3, while all WHO values are provided for comparison. No statistical new testing is presented in this table.

Supplementary Data 9: Genomic positions of genes used in this study. H37Rv v3 was used for calculating positions.

Supplementary Data 10: Interaction coefficients for co-occuring mutations associated with resistance. Interaction coefficients are shown for mutations with significant individual effects that also co-occurred at least 5 times. All MICs are reported in log2 space. Coefficients represent mean effect on MIC in censored interval regression model. Coefficients with two mutations joined by a colon represent the interaction effect. 95% confidence intervals are provided. Mutations that have significant effects (BH_CORRECTED_P<0.05) after Benjamini-Hochberg correction are highlighted in green.
