# Supplementary material for: Quantitative measurement of antibiotic resistance in Mycobacterium tuberculosis reveals genetic determinants of resistance and susceptibility in a target gene approach
Source: Nat Commun. 2024 Jan 12;15:488. doi: 10.1038/s41467-023-44325-5 (PMC10786857; doi:10.1038/s41467-023-44325-5)
Supplement: Supplementary file 5 — Reporting Summary [file 41467_2023_44325_MOESM5_ESM.pdf]

nature portfolio

Corresponding author(s): Joshua Carter

Last updated by author(s): Nov 1, 2023

Reporting Summary

Nature Portfolio wishes to improve the reproducibility of the work that we publish. This form provides structure for consistency and transparency in reporting. For further information on Nature Portfolio policies, see our Editorial Policies and the Editorial Policy Checklist.

Statistics

For all statistical analyses, confirm that the following items are present in the figure legend, table legend, main text, or Methods section.

n/a	Confirmed	
☐	☒	The exact sample size (*n*) for each experimental group/condition, given as a discrete number and unit of measurement
☐	☒	A statement on whether measurements were taken from distinct samples or whether the same sample was measured repeatedly
☐	☒	The statistical test(s) used AND whether they are one- or two-sided<br>*Only common tests should be described solely by name; describe more complex techniques in the Methods section.*
☐	☒	A description of all covariates tested
☐	☒	A description of any assumptions or corrections, such as tests of normality and adjustment for multiple comparisons
☐	☒	A full description of the statistical parameters including central tendency (e.g. means) or other basic estimates (e.g. regression coefficient) AND variation (e.g. standard deviation) or associated estimates of uncertainty (e.g. confidence intervals)
☐	☒	For null hypothesis testing, the test statistic (e.g. *F*, *t*, *r*) with confidence intervals, effect sizes, degrees of freedom and *P* value noted<br>*Give P values as exact values whenever suitable.*
☒	☐	For Bayesian analysis, information on the choice of priors and Markov chain Monte Carlo settings
☒	☐	For hierarchical and complex designs, identification of the appropriate level for tests and full reporting of outcomes
☒	☐	Estimates of effect sizes (e.g. Cohen's *d*, Pearson's *r*), indicating how they were calculated

Our web collection on statistics for biologists contains articles on many of the points above.

Software and code

Policy information about availability of computer code

Data collection	The Clockwork variant calling pipeline is available from: https://github.com/iqbal-lab-org/clockwork.
Data analysis	Scripts used for statistical analysis in StataIC v16 and analysis of results in RStudio are available from: https://github.com/carterjosh/cryptic-mic. A DOI for the Github repo is available here https://zenodo.org/doi/10.5281/zenodo.10065150 2023.

For manuscripts utilizing custom algorithms or software that are central to the research but not yet described in published literature, software must be made available to editors and reviewers. We strongly encourage code deposition in a community repository (e.g. GitHub). See the Nature Portfolio guidelines for submitting code & software for further information.

Data

Policy information about availability of data

All manuscripts must include a data availability statement. This statement should provide the following information, where applicable:

- Accession codes, unique identifiers, or web links for publicly available datasets
- A description of any restrictions on data availability
- For clinical datasets or third party data, please ensure that the statement adheres to our policy

The ENA IDs, VCFs, and MICs are all available here at a permanent and publicly available ftp site at EMBL-EBI (http://ftp.ebi.ac.uk/pub/databases/cryptic/release_june2022/).

Field-specific reporting

Please select the one below that is the best fit for your research. If you are not sure, read the appropriate sections before making your selection.

☒ Life sciences ☐ Behavioural & social sciences ☐ Ecological, evolutionary & environmental sciences

For a reference copy of the document with all sections, see nature.com/documents/nr-reporting-summary-flat.pdf

Life sciences study design

All studies must disclose on these points even when the disclosure is negative.

Sample size	Sample size for each drug was determined by including all isolates for which high quality phenotypes and whole-genome sequences were available.
Data exclusions	Isolates were excluded from analysis if they had a mixed allele call at a previously identified genomic loci associated with resistance or if the phenotype was poor quality (further described in Methods).
Replication	This study provided a descriptive analysis of a large-scale quantitative resistance dataset. The lack of other published large datasets with quantitative resistance data for tuberculosis limits replication of this study currently.
Randomization	Randomization is not relevant to this study. Mixed effect models including a variable for site of collection were used to control for phylogenetic and technical variability.
Blinding	Blinding was not possible for this study as the purpose of the analysis was to link individual genomic mutations with a resistance phenotype.

Reporting for specific materials, systems and methods

We require information from authors about some types of materials, experimental systems and methods used in many studies. Here, indicate whether each material, system or method listed is relevant to your study. If you are not sure if a list item applies to your research, read the appropriate section before selecting a response.

Materials & experimental systems

n/a	Involved in the study	
☒	☐	Antibodies
☒	☐	Eukaryotic cell lines
☒	☐	Palaeontology and archaeology
☒	☐	Animals and other organisms
☒	☐	Human research participants
☒	☐	Clinical data
☒	☐	Dual use research of concern

Methods

n/a	Involved in the study	
☒	☐	ChIP-seq
☒	☐	Flow cytometry
☒	☐	MRI-based neuroimaging
